# Economic Burden of Denatured Alcohol-Induced Burns: A 20-Year Retrospective Study

**DOI:** 10.3389/fmed.2022.914976

**Published:** 2022-06-15

**Authors:** Michela Venturi, Francesco Bruzziches, Catuscia Orlandi, Mattia Altini, Pietro Rubegni, Davide Melandri

**Affiliations:** ^1^Dermatology Unit and Burn Center, Azienda Unità Sanitaria Locale (AUSL) Romagna, Bufalini Hospital, Cesena, Italy; ^2^Dermatology Unit, Department of Medical, Surgical and Neurological Science, Santa Maria alle Scotte Hospital, University of Siena, Siena, Italy; ^3^Medical Direction, Azienda Unità Sanitaria Locale (AUSL) Romagna, Ravenna, Italy

**Keywords:** burns, denatured alcohol, methylated spirits, costs, costs analysis

## Abstract

Burn care has rapidly improved over the past decades, but health innovations are expensive. We present the first study focusing on the economic burden of exclusive denatured alcohol-induced burns. The goal of this study was to determine costs for the public health system due to inpatients’ burn care because of these specific burns. Moreover, we aimed to observe the incidence of methylated spirit-related burns in the past 20 years. We performed an observational retrospective study in our burn unit including all patients with a denatured alcohol-related burn injury from 1 January 2001 to 31 December 2020. A total of 503 patients with a mean burn size of 24% were hospitalized; the mean annual total costs per patient was €43,879, varying from €31,518 to €63,274.00€; the total costs for denatured alcohol-related burns during the period 2001–2020 was €21,145,076. We noted an increasing incidence of denatured alcohol-related burns and related costs over the years, especially in the last decade. Our results highlight that burns by methylated spirits are still a real and expanding problem. Therefore, authorities should focus on sales rules, characteristics of the containers, and education of people who misuse denatured alcohol, based on historical habits of use. To reduce the socioeconomic costs of burns, future intervention strategies and studies from the dermatology community and burn specialists should focus on prevention programs and prompt wound healing to shorten the length of hospital stay, enable quick return to work, and improve the outcomes of patients with burns.

## Introduction

A burn injury is a common type of traumatic injury causing considerable morbidity and mortality. Moreover, burns are also among the most expensive traumatic injuries because of long hospitalization and rehabilitation and costly wound and scar treatments.

Burn care has greatly improved over the past few decades, with the concept of early excision and grafting and a multidisciplinary approach to burn care (1–3), so even patients with extensive burns can survive today (4, 5). Further advances in wound healing, rehabilitation, and counseling are desirable to help burn survivors achieve an optimal quality of life. Unfortunately, health innovations can be very expensive; therefore, cost-effectiveness analysis should also be considered.

Total costs of burn care in high-income countries vary widely across studies; hospital stay and intensive care in burn centers are reported to be the major cost components (6–8), accounting for approximately 82% of total burn care costs per patient, and surgery was another major cost category.

Sometimes, a so devastating trauma for patients and high charges for the healthcare system are due to misuse of trivial substances, such as denatured alcohol (also called methylated spirit), a cheap liquid sold freely to the general population in supermarkets. Denatured alcohol is ethanol with additives, such as methanol (methyl alcohol), that make it bad tasting to discourage drinking. It is commonly used to initiate the ignition of barbecues, fireplaces, campfires, wood-burning stoves, spirit burners, or it can be used during cleaning, or to eliminate insects, or for hairdressing or poured on fires; in rare cases, it could be used for self-inflicted injuries (9–11). Furthermore, the damage caused by denatured alcohol can affect not only users but also close people. The problem of methylated spirit burns has also emerged in the burn literature of previous studies, presenting a casuistry linked to the misuse of accelerants, in particular denatured alcohol (9, 10, 12–14).

## Materials and Methods

Our burn center at M. Bufalini Hospital in Cesena is a referral center for a large territory of the north-middle east of Italy, including Romagna and a part of the Emilia, Umbria, Marche, and Abruzzo regions, but patients from other regions and countries can be transferred in our burn center. It accommodates four intensive care and four sub-intensive care beds.

We retrospectively reviewed our database searching for patients with denatured alcohol burns who were admitted in our burn unit between 1 January 2001 and 31 December 2020. Our referral criteria for inpatients included all children with burns over 5% and adults with burns over 10% of the total body surface area (TBSA), burns in critical areas (i.e., face, hands, genitalia, and perineum), full-thickness burns > 5% of TBSA, and burns with associated inhalation injury. Data regarding patient baseline characteristics and healthcare needs were found in hospital patient records. In particular, for each patient, we noted age, gender, year of injury, mechanism of injury, burned TBSA, need for orotracheal intubation, blood transfusion, length of stay, and, eventually, death.

The national cost rate per burned patient admitted to a burn center has been defined as €1,751 per day in our region (Emilia-Romagna) and in Italy since 2013, either for intensive and sub-intensive burn care. This amount represents a comprehensive reimbursement of costs related to inpatient care, because the cost analysis of this function in previous years showed a remarkable discrepancy between the evaluation of the Diagnosis Related Groups (DRGs) rate activity and the real sustained costs. The remuneration at a specific DRG rate does not correctly grasp the peculiarities of the resource absorption profile of cases related to this discipline. Hence, we applied the most recent cost for everyday hospital stay of the patients included in the study for the whole observed period to assess the cost of hospitalization.

## Results

**FIGURE 1:**
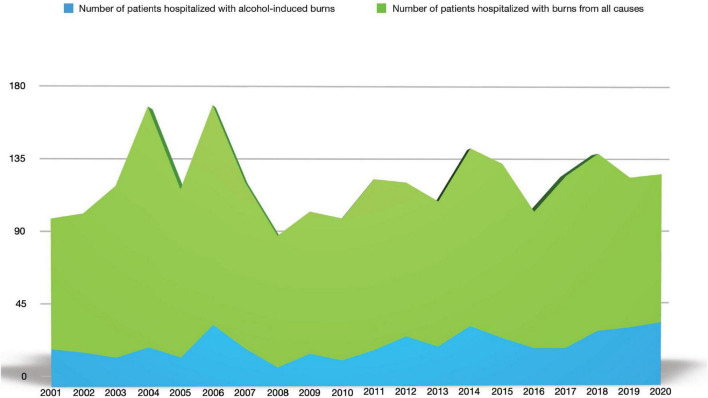
Denatured alcohol-induced burns over all-cause burns in inpatients in our burn center in the period 2001–2020.

A total of 2,286 patients were admitted to our burn unit at M. Bufalini Hospital in Cesena, Italy, between 1 January 2001 and 31 December 2020 because of a burn injury due to any cause. Among them, 503 subjects declared denatured alcohol-related skin lesions and were reviewed for the present observational retrospective study (Figure 1). In particular, 197 patients were found in the period 2001–2010 and 306 in 2011–2020, with an increase in the number of inpatients equaling to 64% in the second decade when compared to the first.

Their mean age was 54 years (range 1–102 years); 53.48% of the patients were male.

The mean denatured alcohol-related burn size was 24% (range 1–95; Table 1), and 10.7% of the patients required orotracheal intubation, 31.4% needed blood transfusion, and 8.3% died during hospitalization.

**TABLE 1:** Sex, age, total body surface area (TBSA) of the examined patients.

Patient data	
Females	46.52%
Mean age	53.7
Mean females	55.4
Mean age males	52.2
Mean TBSA burned	24%
Second-degree burn or more	17%
Median TBSA burned	20
Mode TBSA burned	20
S.D. in TBSA burned	17.01

**FIGURE 2:**
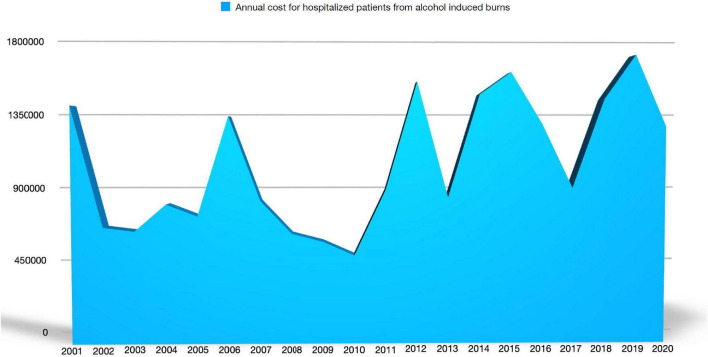
Incidence of denatured alcohol burns in the examined period 2001–2020.

The mean length of stay was 25 days (range 1–250 days). During the complete data analysis period, it was observed that denatured alcohol burns were found to be about 22% of the total burns. Furthermore, comparing the incidence of denatured alcohol burns in the two decades under analysis (2001–2010 and 2011–2020), there was an increase of 10% over the two periods. In the first decade, denatured alcohol burn-related admissions were 17% of the total causes, and in the following decade, this rate increased to 27% (Figure 2).

In our study, the total cost of denatured alcohol burns in the 2001–2020 period was €21,145,076. In the decade 2001–2010, it was €8,233,202, while in the decade 2011–2020 it amounted to €12,911,874, with an increase of 63% in the second decade compared to the first.

**FIGURE 3:**
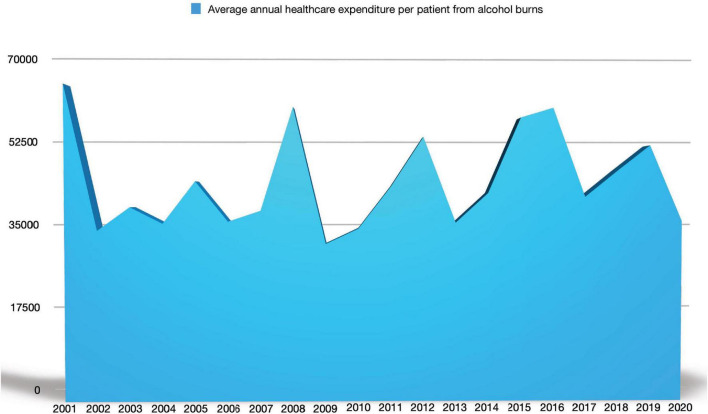
Mean annual total costs per patient injured because of denatured alcohol.

The mean annual total cost per patient was €43,879, varying from €31,518 to €63,274 (Figure 3).

## Discussion

Among health-related problems during the last few decades, burns appear to have some of the most important socioeconomic consequences. Burn treatment has often been identified as expensive, especially compared to other known health problems in high-income countries (15, 16).

Comparing our mean annual cost for denatured alcohol burn victims’ treatment (€43,879) with the mean annual cost of stroke survivors in Italy during the first year after stroke (€11,747) (15) or the mean annual cost of HIV treatment in Italy [€6,399.23 (16)] burns present the highest expense.

According to our knowledge, in the published literature, mean total costs for other patients with trauma were often estimated lower (€10,603–€26,468) than for patients with burns (17).

In a study on intensive care unit (ICU) costs of patients with burn, the authors compared the ICU-associated cost of medications, infusion fluids, laboratory tests, radiological investigations, and physiotherapy sessions of patients with burns with controls matched for length of stay and acuity; they found that ICU-related costs were similar between the groups except for medical imaging, wound dressings, and physiotherapy costs (18). Moreover, the study only considered costs directly indexed to the ICU budget and did not include operative or ward and rehabilitation costs post-ICU discharge (18). Another analysis revealed that for each additional percent of body surface area grafted, hospital costs increased by US$ 2,63919 (19); thus the hospital cost for patients with burns may be higher.

A systematic review showed that the mean cost per patient with burn in high-income countries was US$ 88,218 (17). In particular, the average cost of per patient burn care has been reported as US$ 6,755 in Japan (20), US$ 15,250 in Turkey (21), US$ 73,532 in Australia (22), US$22,759 (€26,540) in the Netherlands (23), US$ 114,576 (€105,116) in the United Kingdom (UK; for a patient with 27% TBSA) (7), US$ 22,617 in the United Kingdom per burn care in primary and secondary care settings (24), US$ 2,283 in Nepal as direct cost for inpatient burn treatment (25), US$ 1,060.5 in North India, US$ 704 in Bangladesh, and US$ 559.85 per patient in Malawi (25). Data were different in high vs. low- and middle-income countries (LMICs; mean costs per patient $88,218 vs. $5,196), as in the last countries there are different healthcare and finance structures, price levels, treatment protocols, and therapeutic possibilities, and limited resources (17, 25). The wide discrepancies in various studies may also be due to differences in the examined sample size, study subjects (children vs. adults), TBSA and burn depth, and differences in methodological approaches, such as bottom-up hospital costing (detailed itemized costing of staff, equipment, drugs, consumables, and maintenance), top-down costing (from detailed budgetary analysis for the hospital), survey-based analyses, and DRGs (26, 27).

The purpose of this study was to focus on assessing the denatured alcohol’s causal role in burn injury and its economic burden to raise the awareness of health authorities about the cost of this product for the national healthcare system and to show the danger of denatured methyl alcohol to the general population. To our knowledge, we present the first study focusing on the economic burden of exclusive denatured alcohol-induced burns caused by its improper and careless use.

In our study, the total costs for denatured alcohol-related burn injuries during the period 2001–2020 was €21,145,076, with average total costs equaling to €43,879 per inpatient. Recent studies on burn costs in high-income countries mirror our findings; they present burn care costs ranging from €6,436 to €73,398 per patient for all etiologies (22, 28–30). In a study from Finland on fire-related burns, the authors found that the median cost for %TBSA was € 2,120 but increased significantly with high %TBSA, and 7–8% of the cases (the extreme and most severe ones) required up to 50% of the total costs (30). Higher age was shown to be associated with higher costs and because our population is aging, in the near future, burn care costs are likely to rise too (26, 30).

The socio-demographic distribution of burn victims is another relevant factor, as groups, such as women, children, and the elderly, require more resources and incur greater costs (10). In our study on denatured alcohol-related burns, the mean age of subjects was 53.7 years, 46.5% were women, and the mean burned TBSA was 24%. The last data are confirmed by a case series reporting severe burns due to biofuel heater injury with an average burned TBSA equal to 24.7% (31). However, these factors differ by country and possibly its socioeconomic level, and by the cause, TBSA, and depth of burns.

Moreover, a burn injury leads to loss of work activity, so extra-hospital costs should also be mentioned. Although the cost for elderly patients is higher in terms of hospital costs for the same TBSA compared to young people (mainly due to comorbidities and later recovery) (30), the loss of work productivity in young people must be considered in the overall cost of denatured alcohol-related burns. Using the human capital approach (that transforms years of life into monetary units) for indirect costs and a bottom-up costing approach, Sanchez et al. (28) reported that the mean annual cost (direct and indirect) per patient with burn in Spain was US$ 99,773, with inpatient care and temporary and permanent disability as the most important expense categories. In particular, direct healthcare costs of patients with burns represented only 19.6% of the total, so costs due to loss of productivity accounted for more than 80%. In the United States, a study estimated that the cost in terms of human capital per medically treated burn injury amounted to US$ 15,733 for acute and long-term treatments, wage loss following injury, and work loss in the event of permanent disability (32).

In addition, a series of submerged health costs should also be considered. One of these is the loss of quality of life of people admitted to burn centers, assessed based on quality-adjusted life-years (QALYs). As pointed out by Miller et al. (33), burns reduce the short-term quality of life by 30% of initial levels, with long-term losses comparable to traumatic brain injury. Globally, it was evaluated that the average person suffering from a fire, heat, or hot substance injury lost 3.2% of their full health status (32). According to the WHO, burns cause more than 7.1 million injuries each year worldwide, loss of almost 18 million disability-adjusted life years (DALYs; approximately 94% of those in LMICs), and more than 265,000 deaths (approximately 92% of those in LMICs) (34).

Sanchez et al. (35) measured the quality of life of patients at the end of the follow-up period using the EuroQol 5-Dimensions survey. The group found that the mean cost per patient, including social and labor costs, was $95,551, with healthcare costs amounting to only 10%, while the labor costs amounted to 56% and, together with the social costs, constituted 85% of the total costs (35). The outcome and returning to work after a burn injury depends on many factors, i.e., predictors of absenteeism could be TBSA, length of stay, ICU admission, surgery, and psychiatric disorders (36).

At the extreme end, 8.3% of the patients in our casuistry died during hospitalization, not so far from a mortality rate study that found that about 5% of hospitalizations from burns ended in hospital death (37), quite similar to the fire-related burn study from Finland where 6% of the patients died during care (30). Accelerant-related burn-injured patients have a mortality rate 3 times higher than that of other burn victims (27). The indirect monetary burden of burn deaths is also considerable. Haikonen et al. (38), using the human capital method, found that the total productivity loss in Finland in the period 2000–2010 was c.a. € 342 million, an annual average of € 31.1 million, with the mean for a victim being €0.315 million. In addition to monetary losses, some 30,000 years of life were lost because of premature deaths (38).

In our study, we found that denatured-alcohol burns were about 22% of the total causes with an increasing incidence of 10% in the two analyzed decades (17% in 2001–2010 and 27% of all etiologies in 2011–2020). This incidence trend has led to raising costs of inpatient care over the years, but our data revealed a surprising augmentation in the economic expenses of up to 63% in the second decade compared to the first, as the costs were €8,233,202 in 2001–2010 and €12,911,874 in 2011–2020. This raising cost mirrors the increased absolute number of inpatients in the two periods, i.e., 197 subjects in the first decade and 306 in the second (+ 64% of patients in the last period with denatured alcohol-related burns). Also, the incidence in our casuistry is impressive, as in the study published by Jansbeken et al. (9) methylated alcohol burns were 3% of the inpatient population. Otherwise, the increasing trend is similar to other studies that pointed out a relative increase in methylated spirit burns over the years (9). This can be explained by increasing outdoor recreational activities in family groups, such as reunions for barbecues or campfires, with fewer preventive measures, or the increasing popularity and distribution of burn devices. Even in the past coronavirus disease 2019 (COVID-19) pandemic years, an increasing incidence of denatured alcohol-linked burns was observed, due to home quarantine, the practice of hand sanitizing using alcohol, as well as family recreation (12). Accidents due to denatured alcohol also occur because it burns with an invisible flame under bright light (13, 14). Moreover, it is often thrown onto already ignited fires, so the liquid ignites, the flame follows the stream of fuel into the bottle, and the inside vapor with the whole bottle explodes, propelling burning liquid at the user (9, 13, 14). An important safety preventive measure could be a flame arrester that manufacturers could install in the bottle. That would prevent the explosion, blocking the flame from following the stream of liquid into the bottle (13). Moreover, it was observed that a dispenser with a liquid jet opening less than about 0.9 mm does not propagate the flame back toward the vapor inside the container, limiting the risk of explosion (14). Another safe option would be production of a diluted form of denatured alcohol to prevent explosion. Finally, many people are not even conscious of the hazard of denatured alcohol’s invisible flame (13, 14) so educational campaigns are encouraged. Also, from an economic perspective, prevention of burns is a “hot topic” and needs attention. This could generate savings in health costs, avert early mortality, and thus support the survivors’ contribution to the economy. Another similar potential source for severe burn accidents is bioethanol-fueled fireplaces for interior home decoration, which were found responsible for severe burn injuries even though safety instructions were followed (27, 31). Because of the great potential risks, some authors evoked the withdrawal from the market of these products to prevent further harm and potential mortality (31), while others recommended the use of safety fuel gel or changing the bottle cap to a secure lock-cap (39).

This study has some limitations. This is a monocentric study, so it has a limited sample size but it is based on a very large cohort of patients covering a period of 20 years. We did not stratify the patients for age, TBSA, and burn depth, and we did not calculate indirect costs, but these data were beyond the scope of our study that was focused on the costs for the healthcare system linked to methylated alcohol burns. In Eldad’s analysis, hospital stay costs of a severely burned person were attributed to salaries for 37.5%, medical and surgical materials for 22%, medicines 7%, nutrition 3.5%, laboratory 14%, blood, and derived products 15% (40). We did not perform this distinction in our analysis, because we applied an overall reimbursement that was considered a comprehensive inpatient cost defined by the Italian healthcare system in 2013. Besides evolving technologies and standards of care over the years, we also applied this criterion for the previous decade, because this amount is still ongoing and it was calculated by the national healthcare system on previous DRGs and real costs. Hence, it should also reflect the overall expenses of the past years.

## Conclusion

The present article, the first focusing exclusively on the financial burden of denatured alcohol burns, is supposed to increase attention to the hazard of the use of denatured alcohol and the relative economic costs of its care. Furthermore, there are non-care (indirect and/or intangible) costs to be evaluated, such as rehabilitation costs and considerable human, financial, and time resources, required to the patient and his family. The results of our local burn center sited in the north-middle east of Italy could be extended to be representative of the economic burden of denatured alcohol-related burn injuries all over the country and in other high-income countries.

Most methylated spirit burns are preventable, so the best care for these burn injuries is prevention. As a further improvement in burn management, prevention of burn injury is crucial to decrease the long-term disability, morbidity, mortality, and economic burden caused by severe burns. Law and regulation, environmental and consumer product designs, and educational programs are landmarks in prevention (41). Hence, the results of this study should support and stimulate new studies and strategies on the prevention of denatured alcohol burns, new health policies of public awareness, and encourage the correct use of methyl alcohol in the general population.

Further burn care cost studies should focus on long-term economic expenditures; topics to explore are the extent of skincare costs (such as dermatologic medications for residual disepithelialized areas, reconstructive surgery, and scar treatments), medical costs not related to the burn center (such as rehabilitation), long-term non-medical costs (such a permanent absence from work), and the significant impact on quality of life and stress disorders in patient subgroups and families (42). Additionally, to reduce the costs of burn care, future intervention studies from the dermatology community as well as burn specialists should focus on timely wound healing to reduce the length of hospital stay and enable a quick return to work. National and international registrations of burn injuries will enable further epidemiologic research and will certainly lead to better targeted prevention programs and campaigns and cost-effective multidisciplinary burn treatments. Dermatologists, as specialists in skin failures, such as burn injuries, are part of this multidisciplinary burn team and should know the costs of burns and should be involved in prevention programs, future studies, and strategies to improve wound healing and burn patient outcome.

## Data Availability Statement

The raw data supporting the conclusions of this article will be made available by the authors, without undue reservation.

## Ethics Statement

Ethical review and approval was not required for the study on human participants in accordance with the local legislation and institutional requirements. Written informed consent for participation was not required for this study in accordance with the national legislation and the institutional requirements.

## Author Contributions

All authors listed have made a substantial, direct, and intellectual contribution to the work, and approved it for publication.

## Conflict of Interest

The authors declare that the research was conducted in the absence of any commercial or financial relationships that could be construed as a potential conflict of interest.

## Publisher’s Note

All claims expressed in this article are solely those of the authors and do not necessarily represent those of their affiliated organizations, or those of the publisher, the editors and the reviewers. Any product that may be evaluated in this article, or claim that may be made by its manufacturer, is not guaranteed or endorsed by the publisher.
